# Quantitative Ubiquitinomics Revealed Abnormal Ubiquitinated ATP7A Involved in Down-Regulation of ACTH in Silent Corticotroph Adenomas

**DOI:** 10.3389/fendo.2022.863017

**Published:** 2022-05-11

**Authors:** Sida Zhao, Yue He, Hongyun Wang, Dan Li, Lei Gong, Yazhuo Zhang, Chuzhong Li

**Affiliations:** ^1^ Beijing Neurosurgical Institute, Capital Medical University, Beijing, China; ^2^ Beijing Institute for Brain Disorders Brain Tumor Center, Capital Medical University, Beijing, China; ^3^ Beijing Tiantan Hospital, Capital Medical University, Beijing, China

**Keywords:** silent corticotroph adenomas, ATP7A, ubiquitination, mass spectrum, ACTH

## Abstract

Ubiquitination is reported to be a critical biological event on ACTH secretion in corticotroph adenomas. However, the effect of ubiquitylation on ACTH secretion in silent corticotroph adenomas (SCAs) remains unclear. The aim of our study was to explore the mechanism of decreased secretion of ACTH in SCAs with ubiquitinomics. The differently expressed ubiquitinated proteins between SCAs and functioning corticotroph adenomas (FCAs) were identified by 4D label-free mass spectrometer, followed by bioinformatics analysis. The function of the candidate ubiquitinated protein ATP7A (K333) was validated in AtT20 cells. A total of 111 ubiquitinated sites corresponding to 94 ubiquitinated proteins were typically different between SCAs and FCAs. Among all the ubiquitinated sites, 102 showed decreased ubiquitination in SCAs, which mapped to 85 ubiquitinated proteins. Pathway enrichment analysis revealed that ubiquitinated proteins were mainly enriched in vesicle pathway and protein secretion pathway. ATP7A (K333) was one of the proteins enriched in vesicle pathway and protein secretion pathway with decreased ubiquitination level in SCAs. *In vitro* assay indicated that both ATP7A siRNA and omeprazole (ATP7A protein inhibitor) increased the secretion of ACTH in AtT20 cell supernatant compared to control groups (p<0.05). These results indicated that ATP7A might be related to the abnormal expression of ACTH in SCAs and potential for the treatment of SCAs.

## Introduction

Silent Corticotroph Adenoma (SCA) is a kind of high-risk pituitary adenoma. It composed of 20% of all corticotroph adenomas (1). SCAs often exhibit characters of highly proliferative, and up to 43% of SCAs exihibit an aggressive growth into cavernous sinus (2) which lead to a more difficult surgery treatment. Additionally, SCAs patients showed a character of high recurrence rate (nearly 36%) (3) which makes surgery of SCAs difficult. Accordingly, an alternative method is required for a better treatment of SCAs. In the search to establish a reasonable treatment strategy, some scientists have struggled to enlighten the biological mechanism of abnormal blood ACTH level in SCA patients in the past few years.

Different from FCAs patients with Cushing’s Diseases, SCAs patients have no clinical and biological features of Cushing’s Diseases and they show lower blood ACTH level (4). The clinical and endocrinological appearances of SCAs are more like non-functioning pituitary adenomas (NFPAs). Several studies propose hypotheses about the silencing mechanism of SCAs. Kovacs et al. proposed that a large number of lysosomes fused with ACTH secretory granules and since contribute to the damage of secretory granules before release (5). Others suggest that SCAs mainly secret biological inactive ATCH instead of normal ACTH (1–39) (6). Single cells in SCAs may secret ACTH insufficiently or inactively (7).

Ubiquitination is a kind of reversible post-tanslational modification (PTMs) that deeply reported (8). Ubiquitination process is a cascade response regulated by ubiquitin activating (E1), conjugating (E2), and ligating (E3) enzymes, and the ubiquitin molecule (76 amino acids, 8.5Kd) was attached to the lysine residue of the substrate proteins (9). This process controls the dynamic balance of proteins synthesis and degradation. Ubiquitination leads to the degradation of proteins by ubiquitin-proteasome system (UPS) (10). Disfunctional ubiquitination of protein relates to many cell process, such as intracellular trafficking, enzymatic activity regulation and assembly of multiprotein complexes (11). Sesta A et. announced that inhibition of the ubiquitin-proteasome pathway increased ACTH secretion in corticotroph adenomas (12). However, there are no clear reports about the expression profiling of ubiquitination in SCAs and the potential mechanism of ubiquitination in ACTH secretion.

In this study, we clarified the ubiquitinomics in SCAs by 4D mass spectrometer and identified the ubiquitinated proteins which may play important roles in regulating the secretion of ACTH in SCAs. These findings may be meaningful and provide a target for the treatment of SCAs.

## Materials and Methods

### Human Samples Collection

Tumor samples used in this study were obtained from Beijing Tiantan Hospital by transsphenoidal surgery. Fresh tumor samples were stored in liquid nitrogen. Five SCA tissue samples and five functioning corticotroph adenoma (FCA) were used for 4D leble free mass spectrometer. All tumor samples were classified according to the 2017 WHO classification.

This study was approved by the ethics committees of Beijing Tiantan Hospital (KY2018-053-02). Informed consent was obtained from all enrolled subjects, and the study was performed in full compliance with all principles of the Declaration of Helsinki.

### Protein Extraction

The sample was grinded with liquid nitrogen into cell powder and then transferred to a 5-mL centrifuge tube. After that, four volumes of lysis buffer (8 M urea, 1% protease inhibitor cocktail) was added to the cell powder, followed by sonication three times on ice using a high intensity ultrasonic processor (Scientz). (Note: For PTM experiments, inhibitors were also added to the lysis buffer, e.g. 3 μM TSA and 50 mM NAM for acetylation, 1% phosphatase inhibitor for phosphorylation). The remaining debris was removed by centrifugation at 12,000 g at 4°C for 10 min. Finally, the supernatant was collected and the protein concentration was determined with BCA kit according to the manufacturer’s instructions.

### Trypsin Digestion

For digestion, the protein solution was reduced with 5 mM dithiothreitol for 30 min at 56°C and alkylated with 11 mM iodoacetamide for 15 min at room temperature in darkness. The protein sample was then diluted by adding 100 mM TEAB to urea concentration less than 2 M. Finally, trypsin was added at 1:50 trypsin-to-protein mass ratio for the first digestion overnight and 1:100 trypsin-to-protein mass ratio for a second 4 h-digestion. Finally, the peptides were desalted by C18 SPE column.

### Pan-Antibody-Based PTM Enrichment

To enrich modified peptides, tryptic peptides dissolved in NETN buffer (100 mM NaCl, 1 mM EDTA, 50 mM Tris-HCl, 0.5% NP-40, pH 8.0) were incubated with pre-washed antibody beads (Lot number xxx, PTM Bio) at 4°C overnight with gentle shaking. Then the beads were washed for four times with NETN buffer and twice with H2O. The bound peptides were eluted from the beads with 0.1% trifluoroacetic acid. Finally, the eluted fractions were combined and vacuum-dried. For LC-MS/MS analysis, the resulting peptides were desalted with C18 ZipTips (Millipore) according to the manufacturer’s instructions.

### 4D Mass Spectrometer

The tryptic peptides were dissolved in solvent A (0.1% formic acid, 2% acetonitrile/in water), directly loaded onto a home-made reversed-phase analytical column (25-cm length, 75/100 μm i.d.). Peptides were separated with a gradient from 6% to 24% solvent B (0.1% formic acid in acetonitrile) over 70 min, 24% to 35% in 14 min and climbing to 80% in 3 min then holding at 80% for the last 3 min, all at a constant flow rate of 450 nL/min on a nanoElute UHPLC system (Bruker Daltonics).

The peptides were subjected to capillary source followed by the timsTOF Pro (Bruker Daltonics) mass spectrometry. The electrospray voltage applied was 1.60 kV. Precursors and fragments were analyzed at the TOF detector, with a MS/MS scan range from 100 to 1700 m/z. The timsTOF Pro was operated in parallel accumulation serial fragmentation (PASEF) mode. Precursors with charge states 0 to 5 were selected for fragmentation, and 10 PASEF-MS/MS scans were acquired per cycle. The dynamic exclusion was set to 30 s.

### Database Search

The resulting MS/MS data were processed using MaxQuant search engine (v.1.6.15.0). Tandem mass spectra were searched against the human SwissProt database (20422 entries) concatenated with reverse decoy database. Trypsin/P was specified as cleavage enzyme allowing up to 2 missing cleavages. The mass tolerance for precursor ions was set as 20 ppm in first search and 5 ppm in main search, and the mass tolerance for fragment ions was set as 0.02 Da. Carbamidomethyl on Cys was specified as fixed modification, and acetylation on protein N-terminal and oxidation on Met were specified as variable modifications. FDR was adjusted to < 1%.

### Cell Culture and Hormone Measurement

AtT20 cells were purchased from American Type Culture Collection (ATCC; Manassas, VA, USA) and were cultured in F12K medium (ATCC; Manassas, VA, USA) supplemented with 2.5% fetal bovine serum (FBS; Gibco) and 15% horse medium (Gibco).

Cells were plated into 24-well dishes with 200,000 cells and 1ml of medium per well and incubated for 72h. AtT20 cells treated with omeprazole (HY-B0113A, MedChemExpress) and the ACTH levels were assessed by an enzyme-linked immunosorbent assay (ELISA) (SBJ-M0483, SBJbio) according to the manufacture’s protocol.

### Transfection and RNA Interference

siRNA transfection was performed using Lipofectamine 3000 (L3000001, Thermo Fisher), according to the manufacturer’s protocol. siRNA synthesis was performed by Beijing Syngentech and the siRNA sequences for mouse ATP7A is 5’GAACAUGAGUAAUGAAGAAT-T3’.

### Bioinformatic and Statistical Analysis

UbiBrowser (13) was used to generate known and predicted human ubiquitin ligase (E3)-substrate interaction (http://ubibrowser.ncpsb.org.cn). Functional annotation databases were utilized based on the biological process, molecular function, and cellular component classifications of differently expressed ubiquitinated proteins between SCAs and FCAs as determined by Gene Ontology (GO) (available online at http://www. geneontology.org). The enrichment pathway analysis of differently expressed ubiquitinated proteins between SCAs and FCAs was performed based on the Hallmark Gene Sets of Molecular Signatures database (Gene Set Enrichment Analysis, GSEA, http://software.broadinstitute.org/gsea/msigdb/index.jsp). STRING was used to build (protein-protein interactions) PPI networks with the ubiquitinated proteins.

All statistical analyses were conducted using the GraphPad Prism software package (GraphPad Software, San Diego, CA 92108). Unpaired Student’s *t*-tests and chi-squared (Fisher’s exact) tests were used for comparisons of quantitative and qualitative data, respectively. Differences with a *p* < 0.05 were considered significant.

## Results

### Profiling of Ubiquitination Sites in SCA

**Figure 1:**
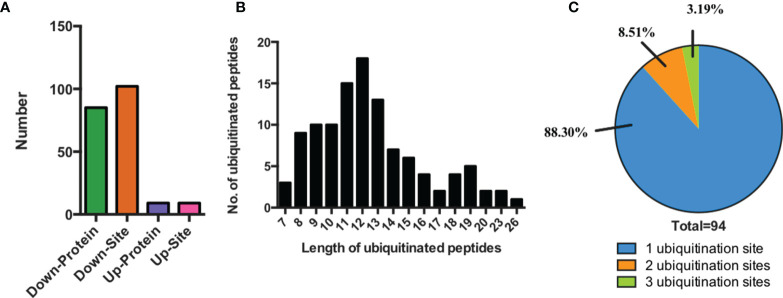
Characteristics of the identified ubiquitinated peptides between SCAs and FCAs. **(A)** The number of differently expressed ubiquitinated peptides and proteins. **(B)** Distribution of ubiquitinated peptides with different length. **(C)** Distribution of ubiquitinated proteins based on the number of ubiquitination sites.

In the profiling result, we identified 111 lysine-ubiquitinated sites totally between SCAs and FCAs (score>40, p>0.05 and fold change>1.5, **Supplementary Table 1**). The 111 lysine-ubiquitinated sites includes 102 down-regulated and 9 up-regulated sites in SCAs. 111 ubiquitinated sites were mapped to 94 proteins, which includes 85 down-regulated and 9 up-regulated proteins in SCAs (**Figure 1A**). The 111 ubiquitinated sites mapped to 110 unique peptides, and only 1 peptide had 2 ubiquitinated sites. The lengths of ubiquitinated peptides were obviously different (**Figure 1B**). For example, the identified ubiquitinated peptides with 11, 12 or 13 amino acid were separately 13.51% (15/111), 16.22%(18/111), and 11.71% (13/111). Meanwhile, most of the identified proteins had single ubiquitination site (88.30%, 83/94) and 8 proteins get 2 ubiquitination site (8.51%, 8/94) and 3 proteins had at least 3 ubiquitination sites (3.19%, 3/94, (**Figure 1C**).

**Figure 2:**
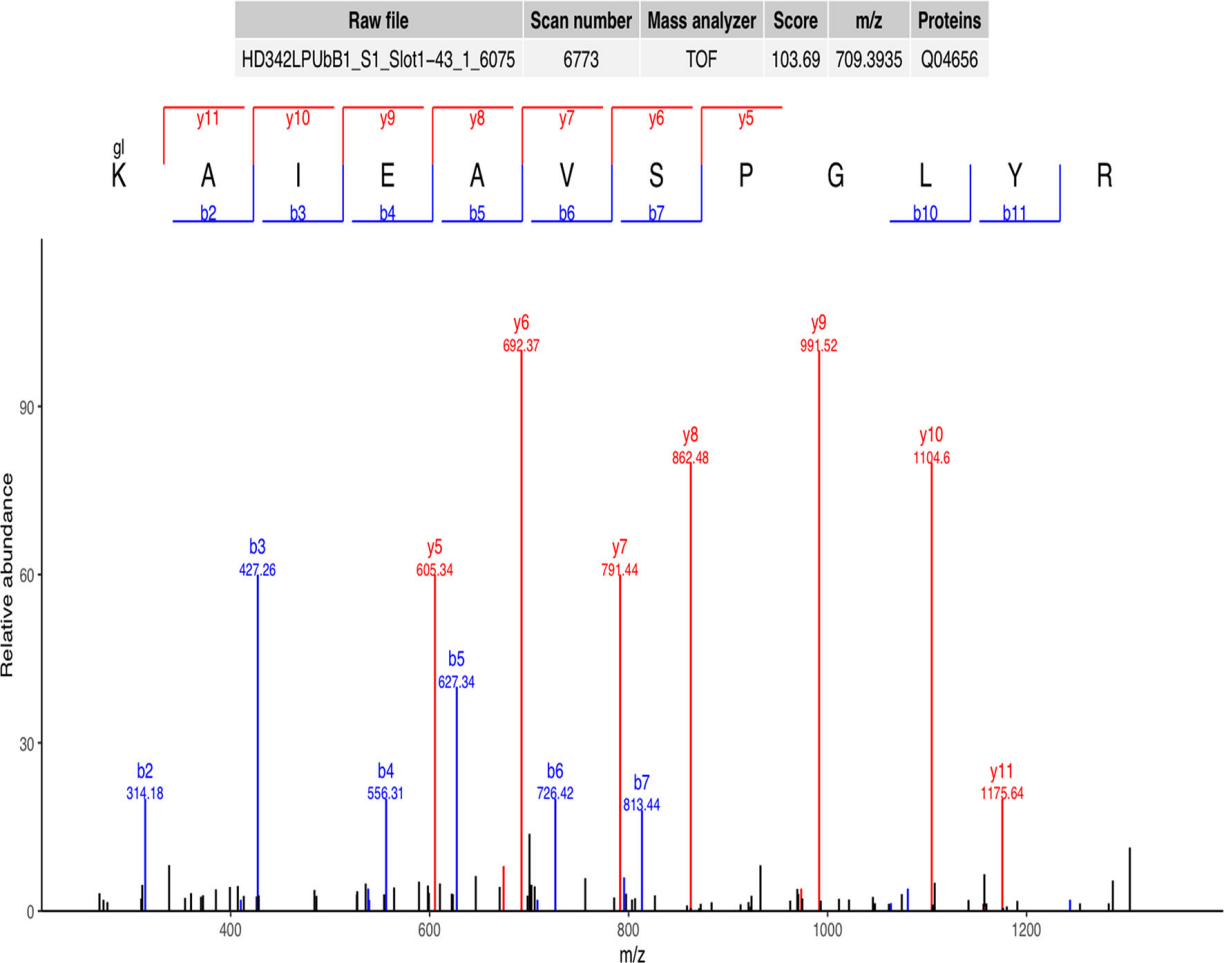
Representative ubiquitinated peptide spectrum: ^333^K^*^AIEAVSPGLYR^344^ (m/z=709.39) from ATP7A (Protein Session: Q04656), K^*^ = ubiquitinated lysine residue.

A representative ubiquitinated peptide spectrum from ATP7A (Protein Session: Q04656) was shown: ^333^K*AIEAVSPGLYR^344^ (m/z=709.39), was a high-quality spectrum, with excellent b ion and y ion series (b2, b3, b4, b5, b6, b7, b10, b11, y5, y6, y7, y8, y9, y10, y11). The ubiquitination site of ATP7A was localized at K^333^ residue, and the ubiquitination level was significantly decreased in SCAs compared to FCAs (ratio of S/F=0.17, **Figure 2**).

### Ubiquitination Motifs Analysis in SCA

**Figure 3:**
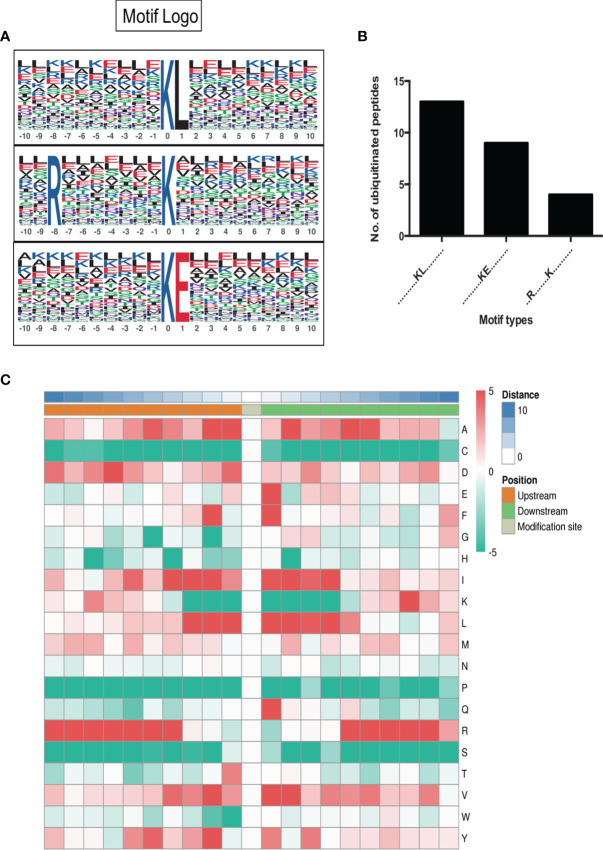
Motif analysis of differently expressed ubiquitination sites. **(A)** Potential ubiquitin recognition motif logos. The height of each letter represents the frequency of the residue in that position. K in the middle represent for the ubiquitinated lysine. **(B)** The number of identified peptides among the three motifs. **(C)** The heatmap for the amino acid distribution flanking ubiquitination sites.

Motif-X was used for analysis of the significant ubiquitination motifs that were preferred to be ubiquitinated in SCAs. We identified 3 significant motifs that were prone to be ubiquitinated in the tumor, including K^ub^-L, K^ub^-E, and R-X-X-X-X-X-X-X- K^ub^ (K^ub^ represent for the ubiquitinated lysine residue and X represents for the amino acid), with 13, 9 and 4 ubiquitinated sites respectively among all of the 111 differently expressed ubiquitinated sites (**Figure 3**). Of them, K^ub^-L was the most significant motif to be ubiquitinated and leucine residue (L) was a vital downstream ubiquitation site. Arginine residue (R) was a representative upstream amino acid for the ubiquitination at K residue. The above 3 types of modification account for 23.42% of all the identified ubiquitinated site in SCAs and FCAs (26/111).

### Functional Enrichment Analysis of the Identified Ubiquitinated Proteins

**Figure 4:**
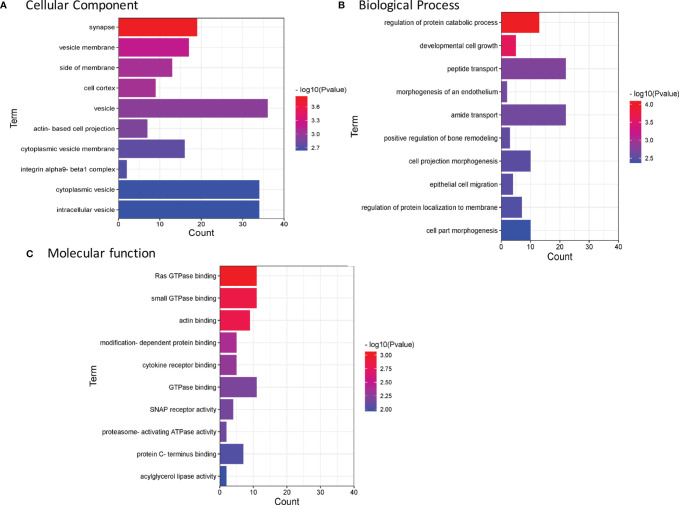
Enrichment analysis of GO annotations in identified ubiquitinated proteins (top 10). **(A)** Cell component. **(B)** Biological process. **(C)** Molecular function. GO, Gene Ontology.

GO enrichment analysis was used to identify the functional clusters of 94 ubiquitinated proteins. Comprehensive analysis revealed that these proteins were obviously involved in many vesicle-related cellular component (**Figure 4A** and **Supplementary Table 2**), such as vesicle membrane (*p=6.46E-04*), vesicle (*p=1.14E-03*), cytoplasmic vesicle membrane (*p=1.77E-03*), cytoplasmic vesicle (*p=2.28E-03*), and intracellular vesicle (*p=2.30E-03*), which may indicate the ubiquitinated proteins were deeply involved in cell storage transport and secretion functions. Besides, ubiquitinated proteins were found to be enriched in multiple biological process (**Figure 4B**) including regulation of protein catabolic process (*p=8.19E-05*), developmental cell growth (*p=2.55E-04*), morphogenesis of an endothelium (*p=2.20E-03*), cell projection morphogenesis (*p=3.11E-03*), epithelial cell migration (*p=3.17E-03*), and cell part morphogenesis (*p=4.28E-03*). The most important molecular functions (**Figure 4C**) were related to Ras GTPase binding (*p=8.64E-04*) and small GTPase binding (*p=1.42E-03*). Menawhile, proteasome-activating ATPase activity pathway (*7.33E-03*) were enriched. Based on the above enrichment analysis result, we concluded that the identified ubiquitinated proteins participate in a variety of biological process and functions.

**Figure 5:**
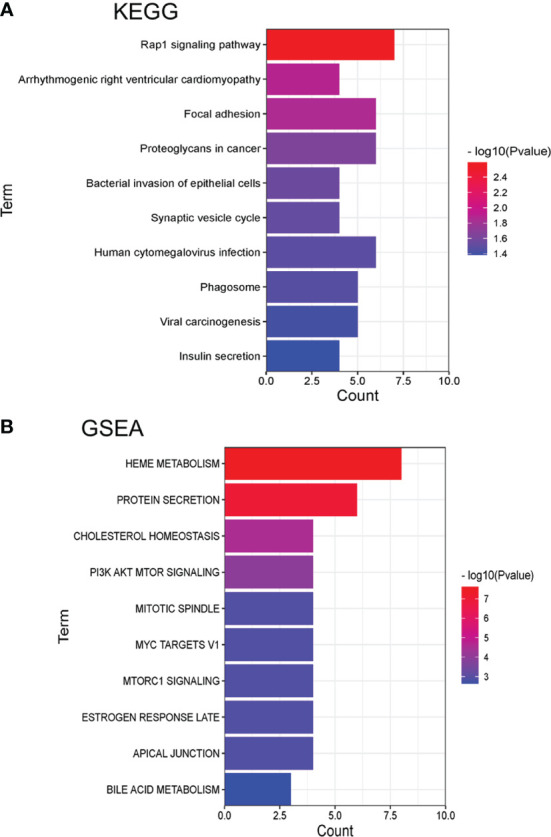
Pathway enrichment analysis of KEGG and GSEA with identified differently expressed ubiquitinated proteins (top 10). **(A)** Pathways by KEGG. **(B)** Pathways by GSEA.

Meanwhile, Kyoto Encyclopedia of Genes and Genomes (KEGG) was used to analyze the pathway enrichment (**Figure 5A** and **Supplementary Table 3**). The result showed that Rap1 signaling pathway (*p= 2.57E-03*), Focal adhesion (*p= 1.39E-02*), Synaptic vesicle cycle (*p= 3.04E-02*) and SNARE interactions in vesicular transport (*p= 4.57E-02*). The above result indicated that ubiquitinated proteins in our profiling involved in a series of fundamental biological process critical for the development of SCA.

Gene Set Enrichment Analysis (GSEA) was also used for the pathway enrichment of the ubiquitinated proteins (**Figure 5B** and **Supplementary Table 4**). The result showed that the most significant pathways were heme metabolism (*p=2.46E-08*) and protein secretion (*p=1.07E-07*) pathway. There were 6 proteins were enriched in the protein secretion pathway, including EGFR, CLTC, ATP7A, AP2S1, RER1, and ERGIC3, indicated that these ubiquitinated proteins were responsible for protein secretion process. The ubiquitinated levels of these proteins were all decreased (**Table 1**). As the table showed that EGFR had 6 ubiquitinated sites while other proteins had 1 ubiquitinated sites.

**Table 1:** Ubiquitinated proteins enriched in protein secretion pathway by GSEA.

Gene name	S/F Ratio	S/F P value	Regulated Type
EGFR	0.32	0.03	Down
EGFR	0.38	0.01	Down
EGFR	0.13	0.00	Down
EGFR	0.30	0.02	Down
EGFR	0.24	0.03	Down
EGFR	0.23	0.01	Down
CLTC	0.39	0.02	Down
ATP7A	0.17	0.05	Down
AP2S1	0.20	0.01	Down
RER1	0.11	0.04	Down
ERGIC3	0.25	0.01	Down

### Molecular Network of Ubiquitinated Proteins in SCAs

**Figure 6:**
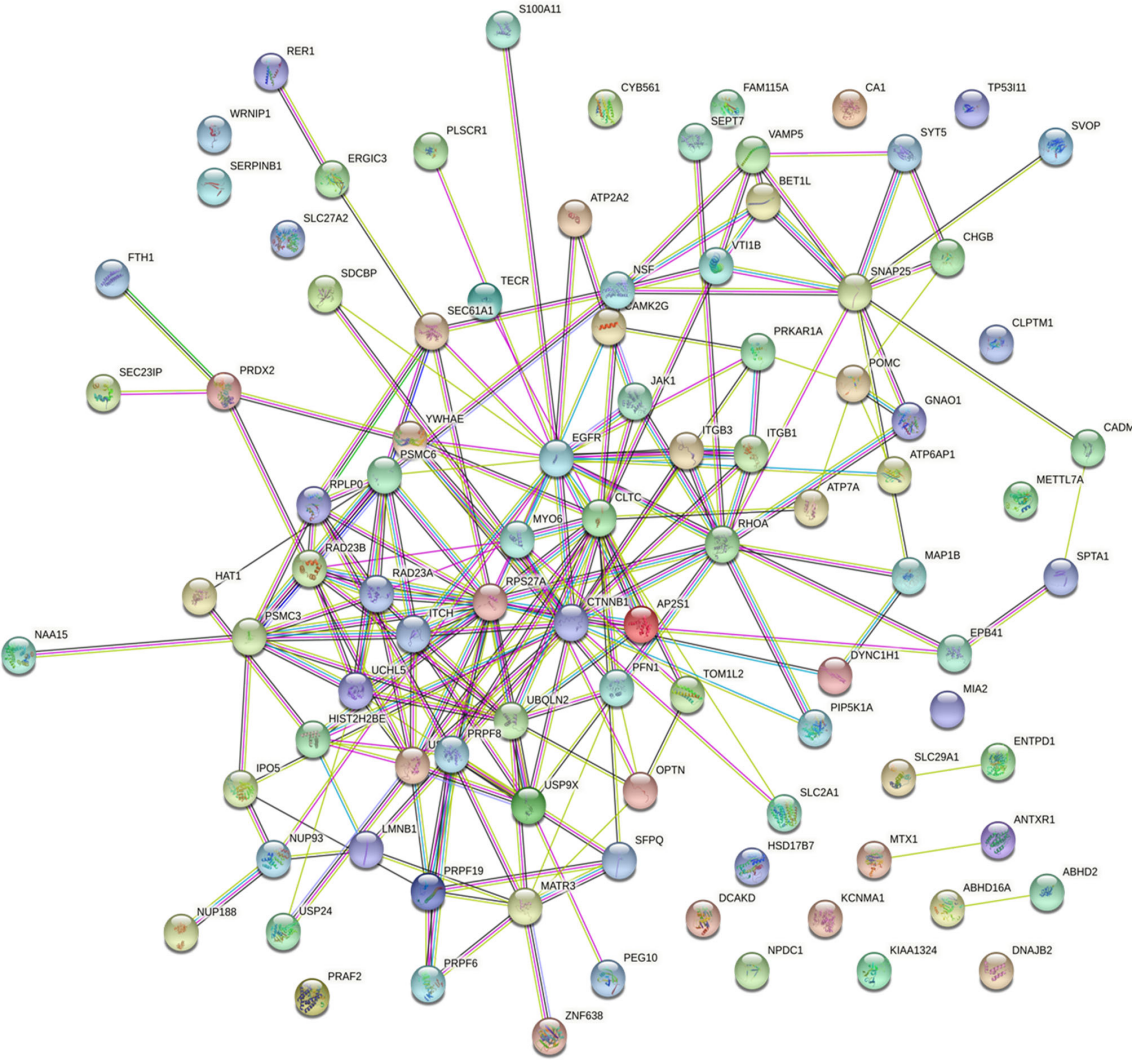
Protein–protein interaction (PPI) network in SCAs.

POMC was an important molecule in corticotroph adenomas and were closely related to the secretion of ACTH, so we explored relation between POMC and ubiquitinated proteins. All of the 94 differently ubiquitinated proteins and POMC were analyzed by STRING database to build PPI networks. There are 95 nodes (proteins) and 195 edges (protein–protein associations) in the network (**Figure 6**). The average node degree and average local clustering coefficient in the network were 4.11 and 0.436, respectively. The PPI enrichment p-value of the network was *4.59E-09*, indicating that ubiquitinated proteins in the network exhibit more interactions among themselves than would be expected for a random set of proteins of similar size obtained from the genome. The network indicated that POMC was interacted with ATP6AP1, ATP7A, CHGB, GNAO1, and PRKAR1A. The combined score of POMC and ATP7A was 0.726 (high confidence, interaction score>0.7, **Table 2**), which was the highest among the 5 POMC related ubiquitinated proteins.

**Table 2:** Proteins interacted with POMC.

#node1	node2	coexpression	database_annotated	automated_textmining	combined_score
POMC	GNAO1	0.062	0.6	0.175	0.663
POMC	ATP7A	0	0	0.726	0.726
POMC	ATP6AP1	0	0	0.508	0.508
POMC	CHGB	0	0	0.427	0.426
POMC	PRKAR1A	0	0	0.602	0.602

### Function of ATP7A in SCAs

**Figure 7:**
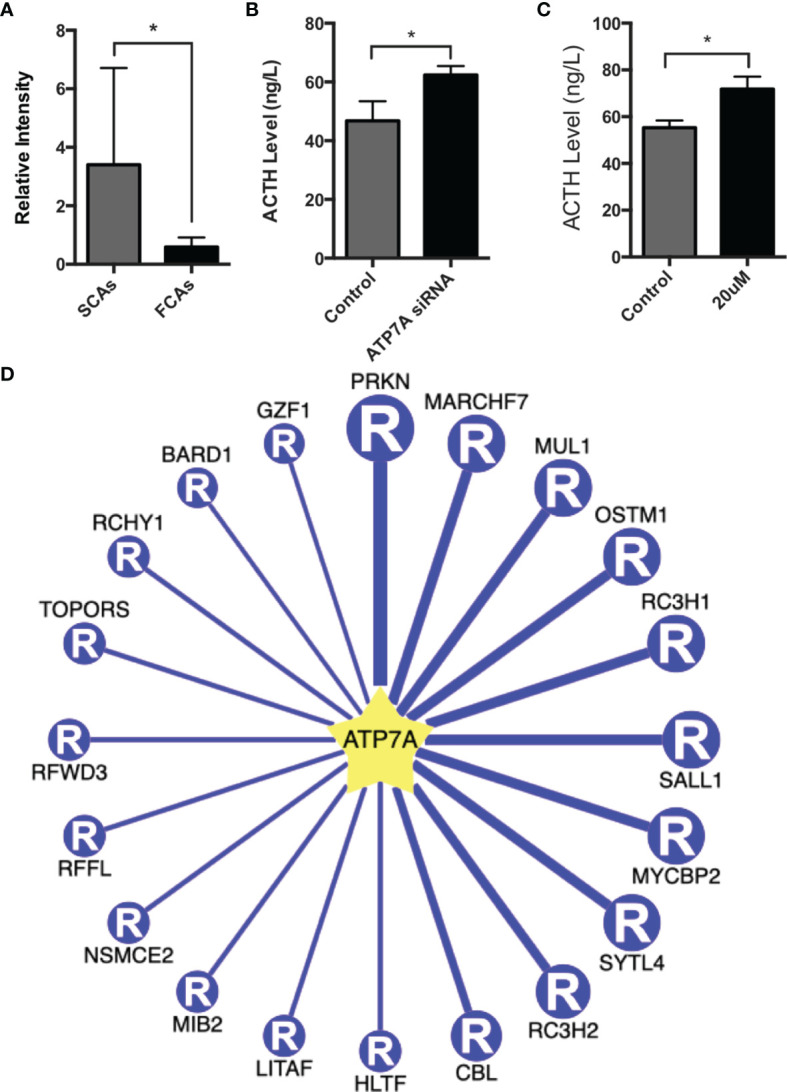
Function of ATP7A in AtT20 cells. **(A)** The intensity of ubiquitinated peptides of ATP7A in SCAs and FCAs. **(B)** Up-regulation of the ACTH level in AtT20 cells with the knockdown of ATP7A by ATP7A siRNA. **(C)** Up-regulation of the ACTH level in AtT20 cells treated with 20uM omeprazole. All assays were performed in triplicate. **(D)** The top 20 potential E3 ligases of ATP7A. *Compared to control, p < 0.05.

ATP7A was ubiquitinated at K333 residue. The ubiquitinated level of ATP7A in SCAs was clearly lower compared to FCAs (ratio of S/F=0.17, *p=0.049*, **Figure 7A**). To validate the function of ATP7A in SCAs, we construct ATP7A siRNA. ATP7A siRNA was transfected to AtT20 cells. ATP7A siRNA result in increased ACTH level in cell culture sodium compared to control groups (**Figure 7B**).

Omeprazole, proved to exert the function of inhibiting ATP7A (14). In our study, omeprazole was used to regulate the expression and detect the function of ATP7A. AtT20 cell was incubated with or without 20uM omeprazole for 72h, and the level of ACTH in culture medium was detected. The result proved that omeprazole increased the expression of ACTH (**Figure 7C**).

38 E3 ligases were predicted to promote the ubiquitination of ATP7A (**Supplemental Table 5**) with high confidence. The top 20 E3 ligases of ATP7A were shown in **Figure 7D**. The top 5 E3 ligases were PRKN, MARCHF7, MUL1, OSTM1, and RC3H1, with the confidence score from 0.85-0.87 and PRKN had the highest confidence score of 0.87. It suggested that PRKN may be a E3 ligases for ATP7A.

## Discussion

Ubiquitination of proteins exhibit a critical role in approximately every biological process (15). This study systematically introduced the ubiquitinome profiling of SCAs. In the study, we identified 94 ubiquitinated proteins with 111 ubiquitinated sites in SCAs compared to FCAs. The pathway enrichment analysis result showed that ubiquitinated proteins were involved in various of biological process, such as vesicle process and protein secretion process. ATP7A, a ubiquitinated protein enriched in vesicle process and protein secretion process, was proved to be able to regulate the expression of ACTH in AtT20 cells. Our findings may help explain the biological behaviors of SCAs and provide promising targets for advanced treatment for SCAs.

Some vesicles are present in neurons or especially endocrine cells including pituitary cells (16), and responsible for the secretion of pituitary hormones (17). Previous studies confirmed that the release of ACTH required vesicles dock with the cell membrane (18). In our study, we concluded that differently expressed ubiquitinated proteins were obviously involved in many vesicle-related pathways, such as vesicle, cytoplasmic vesicle, and intracellular vesicle. On the other hand, GSEA enrichment analysis showed that differently expressed ubiquitinated proteins were enriched in protein secretion pathway. ACTH is a kind of small molecule protein secreted from cells. Taken together, we speculated that these ubiquitinated proteins enriched in vesicle-related pathways and protein secretion pathway may be involved in ACTH release.

Corticaltroph adenomas are immnopositive for ACTH and exhibit with increased circulating ACTH (19). The secretion of ACTH is a flexible process. POMC (Proopiomelanocortin) is a type of polypeptide precursor of neuropeptides and hormones (20). It is cleaved by pro-hormone convertase 1/3 (PC1/3) to yield pro-ACTH and β-LPH (18) in the anterior pituitary. Pro-ACTH was then cleaved by PC1/3 to produce functional ACTH. Meanwhile, ACTH can be cleaved by pro-hormone convertase 2 (PC2) to form α-MSH (21). Different from ACTH, α-MSH need to be amidated by PAM (Peptidylglycine Alpha-Amidating Monooxygenase) enzymes to be active (22). PAM is a kind of peptide-processing enzymes. It catalyzes the post-translational modification of inactive peptidylglycine precursors to the corresponding bioactive alpha-amidated peptides (23). α-MSH is a kind of peptides needs to be amidated and processed by PAM (18). Alpha-amidation involves two sequential reactions, both of which are catalyzed by separate catalytic domains of the enzyme (24). The first step, catalyzed by peptidyl alpha-hydroxylating monoxygenase (PHM) domain, is the copper-, ascorbate-, and O2- dependent stereospecific hydroxylation (with S stereochemistry) at the alpha-carbon (C-alpha) of the C-terminal glycine of the peptidylglycine substrate (25). The second step, catalyzed by the peptidylglycine amidoglycolate lyase (PAL) domain, is the zinc-dependent cleavage of the N-C-alpha bond, producing the alpha-amidated peptide and glyoxylate (26). Several metals are involved in the secretory granule formation of anterior pituitary (27). The accumulation and depot of growth hormone and prolactin in somatotrope and lactotrope secretory granules is separatively promoted by zinc (28). ATP7A (ATPase Copper Transporting Alpha) is a copper tranporter which plays a critical role in intracellular copper ion homeostasis (29). It may supply copper ion to enzyme PAM in the first step (29). In our study, we identified the decreased ubiquitination of ATP7A in SCAs compared to FCAs. Previous researched reported that biquitinated proteins were degraded *via* the ubiquitin-proteasome system (UPS) (30). Thus, we speculated that the decreased ubiquitination of ATP7A in SCAs lead to the increased expression of ACTH compared to FCAs. Since ATP7A is crucial for α-MSH amidation by PAM, the high expression of ATP7A tend to produce more α-MSH and contribute to less production of ACTH. This may explain the decreased blood level of ACTH in SCAs.

Moreover, ATP7A was a crucial ubiquitinated protein enriched in both vesicle-related pathway and protein secretion pathway. It may regulate the ACTH release by protein secretion pathway and vesicle process. There was a study revealed that omeprazole inhibits the function of ATP7A, since ATP7A is a P-type ATPase (14). So we choose omeprazole to block the expreesion of ATP7A in AtT20 cells. *In vitro* assay proved that omeprazole may up-regulate the level of ACTH. PRKN was predicted to be a E3 ligases for ATP7A with a high confidence score. It suggests that ATP7A and PRKN may be potential targets for the treatment of SCAs. ATP7A is a potential ubiquinated protein which was speculated to be a molecule associated with the decreased expression of the ACTH secretion. Ubiquitination is a kind of post-tanslational modification that leads to the degradation of proteins (1). Hence, we speculated that the ubiquitination of ATP7A contribute to the decreased expression of ATP7A. So we used ATP7A siRNA and 20uM omeprazole to change the expression of ATP7A to imitate the function of ubiquitination of ATP7A. However, besides of the function of degradation of proteins, ubiquitination exerts other function on proteins. Here is a limitation that we have not detect the function of ATP7A (K333) in cells. We are now doing this part of work and will report the result in the future study.

4D label-free mass spectrometer is an efficient method to identify differently ubiquitinated proteins and ubiquitination sites in SCAs. Totally 111 differently expressed ubiquitination sites were identified between SCAs and FCAs which mapped to 94 ubiquitinated proteins. Pathway enrichment analysis proved the ubiquitinated proteins were obviously contained in vesicles and involved in protein transport and release. We detected the function of ATP7A in AtT20 cells. Our ubiquitinome profiling result declared the differently expressed ubiquitination sites and proteins in SCAs and FCAs, and also classified the candidate proteins to investigate the hidden mechanism for the decreased expression of ATP7A in SCAs. These discoveries contribute to further researched about the adjustment of ubiquitination in SCAs.

## Data Availability Statement

The original contributions presented in the study are included in the article/**Supplementary Material**. Further inquiries can be directed to the corresponding authors.

## Ethics Statement

The studies involving human participants were reviewed and approved by Ethics committees of Beijing Tiantan Hospital (KY2018-053-02). The patients/participants provided their written informed consent to participate in this study.

## Author Contributions

SZ have drafted the work or substantively revised it. HW, DL, and YH collected tumor samples and analysis the data. LG interpret the data, YZ and CL contribute to the conception of the work. All authors contributed to the article and approved the submitted version.

## Funding

This work is supported by the National Natural Science Foundation of China (82103048, 81771489, 82072804, 82071559).

## Conflict of Interest

The authors declare that the research was conducted in the absence of any commercial or financial relationships that could be construed as a potential conflict of interest.

## Publisher’s Note

All claims expressed in this article are solely those of the authors and do not necessarily represent those of their affiliated organizations, or those of the publisher, the editors and the reviewers. Any product that may be evaluated in this article, or claim that may be made by its manufacturer, is not guaranteed or endorsed by the publisher.
